# Feature Selection for Interpatient Supervised Heart Beat Classification

**DOI:** 10.1155/2011/643816

**Published:** 2011-07-24

**Authors:** G. Doquire, G. de Lannoy, D. François, M. Verleysen

**Affiliations:** ^1^Machine Learning Group, ICTEAM Institute, Catholic University of Leuven, Place du Levant 3, 1348 Louvain-la-Neuve, Belgium; ^2^Neuroscience Institute, Catholic University of Leuven, Avenue Hippocrate 54, 1200 Bruxelles, Belgium

## Abstract

Supervised and interpatient classification of heart beats is primordial in many applications requiring long-term monitoring of the cardiac function. Several classification models able to cope with the strong class unbalance and a large variety of feature sets have been proposed for this task. In practice, over 200 features are often considered, and the features retained in the final model are either chosen using domain knowledge or an exhaustive search in the feature sets without evaluating the relevance of each individual feature included in the classifier. As a consequence, the results obtained by these models can be suboptimal and difficult to interpret. In this work, feature selection techniques are considered to extract optimal feature subsets for state-of-the-art ECG classification models. The performances are evaluated on real ambulatory recordings and compared to previously reported feature choices using the same models. Results indicate that a small number of individual features actually serve the classification and that better performances can be achieved by removing useless features.

## 1. Introduction

 The diagnosis of cardiac pathologies requires monitoring the cardiac function by recording and processing the electrocardiogram (ECG) signal. The diagnosis may rely on just a few transient factors of short duration such as intermittent arrhythmia; long-term ECG recordings are therefore usually required. The manual analysis of such long-term ECG signals, containing hundreds to thousands of heart beats to evaluate, proves tedious and error prone.

Several computer-aided heart beat classification algorithms have been proposed for this task. These algorithms can be divided in two categories: *interpatient* or *intrapatient* classification systems [1]. Intrapatient classification requires labeled beats from the tested patient in the training of the model. By contrast, interpatient models classify the beats of a new tested patient according to a reference database built from data coming from previously diagnosed patients. In real situations, labeled beats are usually not timely available for a new patient which makes the intrapatient classification not applicable. For this reason, this work focuses on interpatient classification.

Supervised classifiers used to automate the classification process require the extraction of discriminative features from the heart beat signals. Spurious features can harm the classifier, especially in the presence of unbalanced classes and a large number of features [2, 3]. Moreover, feature selection serves the interpretability of the classifier, since discriminative features are identified. This property is especially useful in medical applications where the selected features may help to understand the causes and the origin of the pathologies.

Unfortunately, very little information is available to decide how to extract and build features from the heart beat time series. In this work, a large number of features previously proposed for heart beat classification are extracted, and two feature selection methods are investigated to select optimal feature subsets: the wrapper approach using a forward-backward search strategy with a weighted linear discriminant classifier and the filter approach using the mutual information criterion with a weighted support vector machine classifier. Experiments are conducted on real ambulatory signals from the Physiobank arrhythmia database.

The following of this paper is organized as follows.  Section 2 details the state of the art in interpatient classification and emphasizes our contributions to this field.  Section 3 provides a short theoretical background over the classifiers used in this work.  Section 4 reviews methods for feature selection, together with their pros and cons in this particular heart beat classification application.  Section 5 details the database used in the experiments and the processing of the ECG signals.  Section 6 details the experiments and presents the results. Eventually, Section 7 draws some conclusions.

## 2. State of the Art and Contributions

The first study to establish a reliable interpatient classification methodology is [4], where a weighted linear discriminant analysis (LDA) model is trained to classify the beats in the four classes defined by the standards of the AAMI [5]. This algorithm was later improved using the same classifier and other features first in [6] and later by the same authors in [7]. The common point between these algorithms is the use of the weighted LDA classifier, which has three strong limitations. First, it is a linear classifier which will fail to detect nonlinear decision functions. Second, the LDA classifier is based on a Gaussian assumption over class distributions which is not always validated. Finally, the estimation of its parameters becomes difficult in the case of strongly correlated features because of the singularity of the covariance matrix.

For this reason, more powerful classifiers such as support vector machines (SVMs) have also been considered. In [8], hierarchical SVMs are used, but the reported algorithm does not improve the results of [4]. Later, [1] proposed an algorithm based on a support vector machine classifier optimizing a weighted cost function. This algorithm increased the performances of [4] for the pathological classes.

Nevertheless, distinct features groups are considered in each study which makes it difficult to assess their discriminative power on a fair basis. For example, morphological, segmentation, and R-R interval features are considered in [4, 6, 7]. On the other hand, [8] exploited R-R intervals, Hermite basis function expansions, and higher-order statistics. In [1], all these feature sets are considered, but the feature selection is performed only at the group level, without evaluating the relevance of each individual feature included in the classifier.

In this work, our contribution consists in the extraction of all these feature sets and the evaluation of the relevance of each individual feature. For this purpose, a wrapper approach using a forward-backward search strategy and a filter approach using the mutual information criterion are investigated. This is, to our knowledge, the first work (1) evaluating the relevance of all commonly used feature sets on a common ground and (2) using the mutual information criterion to select optimal heart beat features. As it will be detailed later, the mutual information criterion indeed offers many advantages over model-based approaches such as a low computational cost. In the next section, a theoretical background over the weighted LDA and the weighted SVM classifiers is provided, together with an introduction to the mutual information criterion.

## 3. Theoretical Background

 Let us define the *i*th *P*-dimensional observation **x**_*i*_ = {*x*_*i*_^1^, *x*_*i*_^2^,…, *x*_*i*_^*P*^} and the associated class value *y*_*i*_ ∈ {1,2,…, *K*} for a given heart beat *i* with *i* ranging from 1 to *N*, *N* being the total number of heart beats in the dataset and *K* the number of classes. Traditional classifiers optimizing the accuracy make the hidden assumption that the classes are equally balanced [3]. However, in a heart beat classification task, around 90% of beats are normal beats, while all the pathological classes represent the other 10%. For this reason, weights have to be introduced in the classifier to handle that situation. Higher costs are then given to the minority classes so as to guide the training process to solutions which favor these classes. Two distinct models are considered in this work: the weighted LDA model [4, 6, 7] and the weighted SVM model [1].

### 3.1. Weighted LDA

The traditional linear discriminant analysis (LDA) classifier is first described, next it is shown how to adapt its formulation in the case of unbalanced datasets [4]. The LDA approaches the classification problem by assuming that the conditional probability density functions *p*(**x**_*i*_ | *y*_*i*_ = *k*) are normally distributed with the simplifying homoscedastic assumption that the class covariances are identical. All the parameters **w** of the model are thus summarized by the mean class vectors ***μ***_*k*_ and the unique covariance matrix Σ. These parameters are identified by maximizing the log-likelihood function defined as

(1) $$\begin{array}{c} \underset{\mu_{1},\mu_{2},\ldots ,\mu_{K},\Sigma }{\max\;}\;\overset{K}{\underset{k=1}{\sum }}\underset{\left\{i\;|\;y_{i}=k\right\}}{\sum }\log\;\left(f_{k}\left(x_{i},\mu_{k},\Sigma \right)\right), \end{array}$$

where *f*_*k*_(**x**_*i*_, ***μ***_*k*_, Σ) are the value of a Gaussian distribution with mean ***μ***_*k*_ and covariance Σ. The optimization can be done in closed form and yields the following solution:

(2) $$\begin{array}{c} \mu_{k}=\frac{\sum_{\left\{i\;|\;y_{i}=k\right\}}^{}x_{i}}{N_{k}},\\ \Sigma =\frac{1}{N}\overset{K}{\underset{k=1}{\sum }}\underset{\left\{i\;|\;y_{i}=k\right\}}{\sum }\left(x_{i}-\mu_{k}\right){\left(x_{i}-\mu_{k}\right)}^{T}. \end{array}$$

In unbalanced situations, a popular technique is to add distinct class misclassification weights in the objective function. In the case of the LDA classifier, the following decomposition and weighting is applied to the sum over observations in (1) [4]:

(3) $$\begin{array}{c} \underset{\mu_{1},\mu_{2},\ldots ,\mu_{K},\Sigma }{\max\;}\overset{K}{\underset{k=1}{\sum }}c_{k}\underset{\left\{i\;|\;y_{i}=k\right\}}{\sum }\log\;\left(f_{k}\left(x_{i},\mu_{k},\Sigma \right)\right), \end{array}$$

where the *c*_*k*_ parameters are the weights associated to each class. The parameters of the model are now estimated with

(4) $$\begin{array}{c} \mu_{k}=\frac{\sum_{\left\{i\;|\;y_{i}=k\right\}}^{}x_{i}}{N_{k}},\\ \Sigma =\frac{\sum_{k=1}^{K}c_{k}\sum_{\left\{i\;|\;y_{i}=k\right\}}^{}\left(x_{i}-\mu_{k}\right){\left(x_{i}-\mu_{k}\right)}^{T}}{\sum_{k=1}^{K}c_{k}N_{k}}. \end{array}$$

Inference is then achieved using

(5) $$\begin{array}{c} y_{i}^{*}=\underset{k}{\max\;}f_{k}\left(x_{i}\right),\\ f_{k}\left(x_{i}\right)=-\left(\frac{1}{2}\right)\mu_{k}^{T}\Sigma^{-1}\mu_{k}+\mu_{k}^{T}\Sigma^{-1}x_{i}, \end{array}$$

which corresponds to assigning **x**_*i*_ to the class having the smallest Mahalanobis distance between the class mean and **x**_*i*_.

### 3.2. Weighted SVM

A support vector machine (SVM) is a supervised learning method introduced by Vapnik [9]. The two-class case is described here, so *y*_*i*_ ∈ {−1, +1}, because its extension to multiple classes is straightforward by applying the one-against-all or one-against-one approaches. In this work, as detailed in Section 6, the one-against-one approach will be used in the experiments.

SVMs are linear machines that rely on a preprocessing to represent the features in a higher dimension, typically much higher than the original feature space. With an appropriate nonlinear mapping *φ*(**x**) to a sufficiently high-dimensional space, finite data from two categories can always be separated by a hyperplane. In SVMs, the distance from this hyperplane to the nearest data point on each side, referred to as the margin, is maximized. Assume that each observation **x**_*i*_ has been transformed to **z**_*i*_ = *φ*(**x**_*i*_). The soft-margin formulation of the SVM allows examples to be misclassified or to lie inside the margin by the introduction of slack variables *ξ*_*i*_ in the objective constraints

(6) $$\begin{array}{cc} \underset{w}{\min\;} & \;\overset{N}{\underset{i=1}{\sum }}\xi_{i}+\lambda {||w||}_{2}, \end{array}$$

(7) $$\begin{array}{cc} \text{s}.\text{t}. & \;\{\begin{array}{cc} y_{i}\left(\langle w,z_{i}\rangle \right)\geq 1-\xi_{i}, & \forall i=1\cdots N,\\ \xi_{i}\geq 0, & \forall i=1\cdots N, \end{array} \end{array}$$

where **w** are the parameters of the hyperplane. For any feasible solution, misclassified examples have an associated slack value *ξ*_*i*_ greater than 1. We can see from (6) that minimizing the first term minimizes the classification error, while minimizing the second term is equivalent to maximizing the classification margin.

This classical SVM formulation has been shown to suffer from class unbalance and in worst unbalanced cases to yield a classifier biased towards the majority class [10]. The reason is that classifying everything in the majority class is what makes the margin the largest, with zero cumulative loss on the abundant majority examples. The only trade-off is the small amount of cumulative loss on the few minority examples which do not count for much. To overcome this problem, different penalties for each class can be included in the first term of (6),

(8) $$\begin{array}{c} \underset{w}{\min\;}\left(c_{1}\underset{\left\{i\;|\;y_{i}=1\right\}}{\sum }\xi_{i}+c_{-1}\underset{\left\{i\;|\;y_{i}=-1\right\}}{\sum }\xi_{i}\right)+\lambda {||w||}_{2}. \end{array}$$

This weighted formulation of the SVM classifier has been successfully proposed for heart beat classification in [1].

By introducing the Lagrangian multipliers *α*_*i*_, this *primal* formulation can be rewritten in a so-called *dual* form. The optimization is then typically achieved by solving the system using quadratic programming [11]. In the dual form, the explicit form of the mapping function *φ* must not be known as long as the kernel function *K*(**x**_*i*_, **x**_*j*_) = *φ*(**x**_*i*_)*φ*(**x**_*j*_) is defined. The sign of the following decision function is then used to determine the predicted class value *y*_i_* for a new unlabeled observation:

(9) $$\begin{array}{c} y_{i}^{*}=\mathrm{sign}\;\left(f\left(x_{i}\right)\right),\\ f\left(x_{i}\right)=w^{T}\varphi \left(x_{i}\right)=\overset{N}{\underset{j=1}{\sum }}\alpha_{j}y_{j}K\left(x_{j},x_{i}\right). \end{array}$$

### 3.3. Mutual Information

Mutual information (MI) [12] has proven to be a very effective criterion in the context of feature selection, as it is able to detect nonlinear relationships between (groups of) features. The MI value between a given feature and the class labels gives a score over the predictive power of this feature. As an example, in a different area, [13] successfully used MI to determine the most relevant features in spectrometric nonlinear modeling.

Formally, the MI of a pair of random variables *x*, *y* is a symmetric measure of the dependence between these two variables and is defined as

(10) $$\begin{array}{c} I\left(x;y\right)=H\left(x\right)+H\left(y\right)-H\left(x,y\right) \end{array},$$

where *H*(*x*) is the entropy of *x*, which is a measure of the uncertainty on *x*. The entropy is defined for a continuous random variable as:

(11) $$\begin{array}{c} H\left(x\right)=-\int f_{x}\left(\zeta_{x}\right)\log\;f_{x}\left(\zeta_{x}\right)d\zeta_{x}, \end{array}$$

where *f*_*x*_ is the probability density function of *x*. Equation (10) can be written in terms of conditional entropy as

(12) $$\begin{array}{c} I\left(x;y\right)=H\left(y\right)-H\left(y\;|\;x\right) \end{array},$$

where *H*(*y* | *x*) is the conditional entropy of *y* given *x*, measuring the uncertainty about *y* once *x* is known. Following (12), MI can thus be seen as the reduction of uncertainty about *y* brought by the knowledge of *x* and is thus a natural criterion for feature selection assuming that *y* is an output we want to predict from *x*, a set of features.

Eventually, the MI can be expressed as

(13) $$\begin{array}{c} I\left(x;y\right)=\iint f_{x,y}\left(\zeta_{x},\zeta_{y}\right)\log\;\frac{f_{x,y}\left(\zeta_{x},\zeta_{y}\right)}{f_{x}\left(\zeta_{x}\right)f_{y}\left(\zeta_{y}\right)}\;d\zeta_{x}d\zeta_{y}. \end{array}$$

Unfortunately, in practice neither *f*_*x*_, *f*_*y*_ nor *f*_*x*,*y*_ are known. The MI cannot thus be directly computed; it has to be estimated from the available samples. Several methods have been proposed for this task, including a histogram-based estimator [14], a Parzen-window-based estimator [15], and a *k*-NN-based estimator [16]. The MI offers many practical advantages such as the ability to detect nonlinear relationships between the variables and the labels, the use of multiclass labels, and a low computational complexity.

## 4. Feature Selection

Feature selection is traditionally achieved either by wrapper or filter approaches [17]. Wrapper approaches are based on the accuracy of a specific classifier. As an example, the exhaustive wrapper consists in feeding a model with the 2^*P*−1^ possible feature subsets (*P* being the total number of features) and to choose the one for which the model performs the best. This strategy is therefore the optimal feature selection technique for a given model. However, such an exhaustive search is intractable in practice since it would require the training (including the time-consuming optimization of potential hyperparameters) of 2^*P*−1^ different models.

When simple and fast (e.g., linear) models are considered, one can nevertheless circumvent this issue by using an *incremental* wrapper approach [18]. One of the most common incremental search procedures is the forward-backward selection algorithm. Its principle is to select at each step the feature whose addition to the current subset leads to the highest increase in prediction performances. Then it is checked if the removal of one of the previously selected features allows to increase the performances of the model. More precisely, the procedure usually begins with the empty set of features. The first selected feature is then the one which individually maximizes the performances of the model. The second step consists in finding the feature from the feature set which leads to the best increase in performance when combined to the previously selected feature. The procedure continues, but from the third step, a backward step is added to possibly remove a feature if this makes the model perform better. The algorithm is ended when no feature can increase the performance anymore or when a fixed number of features have been reached.

Although this incremental search is not guaranteed to converge to the selection of the optimal subset of features, it has been proven to be very efficient in practice and reduces the required number of models to train from 2^*P*−1^ to *O*(*P*) [19]. Since the training of the weighted LDA model does not require the estimation of any hyperparameter and has a closed-form solution, it only takes a few seconds on a modern computer. Hence, a wrapper algorithm based on a forward search strategy can be used for the weighted LDA classifier. Wrapper approaches, when affordable, are indeed preferred to filter approaches because they are expected to produce better results since they are designed for a specific model.

On the other hand, when it is not affordable to train tens or hundreds of prediction models, feature selection should rather be achieved by filter methods. Filter approaches are based on a criterion independent of the performances of the model (see, e.g., [20, 21]). Those methods are thus much faster than wrapper procedures and are well suited in conjunction with more sophisticated (i.e., nonlinear) models. For example, if the one-against-one approach is used for the multiclass weighted SVM classifier, *P*(*P* − 1)/2 models must be trained for one choice of features, and each model itself requires the tuning of two hyperparameters by leave-one-patient-out cross-validation. To give an idea of the running time, a wrapper forward selection strategy for the weighted SVM model would run in the order of several weeks on a modern computer. Clearly, in such situations, a filter strategy should thus rather be considered.

Since MI is able to detect relationships between random variables and is naturally suitable for multiclass problems, it is a powerful criterion for filter procedures [22, 23]. However, MI can detect nonlinear relationships, and a linear classifier using the given features could possibly fail in grasping the required nonlinear discriminative information. For this reason, only the weighted SVM model with a nonlinear kernel should be tested on the variables selected by the MI ranking procedure. As far as the running time is considered, it only takes a few seconds on a modern computer to estimate the MI value between hundreds of features and the class labels using histograms.

Eventually, is it also worth mentioning that only the computational cost of the feature selection strategy and of the training of the model is to be taken into account, since the computational cost of testing can be achieved in real time for both models.

## 5. Methodology

 Previous work on interpatient heart beat classification uses features extracted from the heart beat signal using either a priori knowledge or by comparing several combinations of feature sets. There is thus a lack of assessment of the relevance of individual features. In this work, two feature selection techniques are investigated to select the individual features serving the classification task. A large number of features are considered and compared on a fair basis. This section introduces the methodology followed in our experiments.

### 5.1. ECG Data

The standard MIT-BIH arrhythmia database [24] is used in the experiments. It contains 48 half-hour-long ambulatory recordings obtained from 48 patients, for a total of approximatively 110.000 heart beats manually labeled into 15 distinct beat types. According to the AAMI standards, the four recordings including paced beats are rejected for a total of 44 experimental recordings [5].

For each recording, two signals from two distinct leads are available. The sampled ECG signals are first filtered using the same filtering procedure as in [1, 4, 8] to remove unwanted artifacts such as baseline wanderings due to respiration, power line interference, and other highfrequency artifacts.

The 44 available recordings are divided in two independent datasets of 22 recordings each with approximatively the same ratio of heart beats classes [4]. The first dataset is the training set and is used to build the model. The second dataset is the test set and is used to obtain an independent measure of the performances of the classifier.

The R spike annotations provided with the database are used as a marker to separate and identify the beats. The MIT-BIH heart beat-labeled types are then grouped according to the AAMI recommendations into four more clinically relevant heart beat classes (see Table 1 for grouping details).  Table 2 shows the number of beats in each class and their frequencies in the two datasets.

### 5.2. Feature Extraction

The popular feature groups previously proposed for heart beat classification are extracted from the heart beat time series: R-R intervals (used in almost all previous works), segmentation intervals [4, 25], morphological features [4, 26], Hermite basis function expansion coefficients (HBF) [8, 27, 28], and higher-order statistics [8, 29]. The following of this section describes the features included in each of the groups.

(1) Segmentation intervals (24 features): the ECG characteristic points, corresponding to the onset and offset of P, QRS, and T waves, are annotated using the standard *ecgpuwave *(see http://www.physionet.org/physiotools/software-index.shtml) segmentation software provided with the MIT-BIH arrhythmia database. A large variety of 24 features are then computed from the annotated characteristic waves:
  - QRS wave: flag, area, maximum, minimum, positive area, negative area, standard deviation, skewness, kurtosis, length, QR length, and RS length;
  - P wave: flag, area, maximum, minimum, and length;
  - T wave: flag, area, maximum, minimum, length, QT length, and ST length.
When the characteristic points needed to compute a feature failed to be detected in the heart beat annotation step, it has been chosen in this work to set the feature value to the patient's mean feature value rather than discarding the beat. Note that only a very small portion of the beats failed to be annotated (e.g., the Q and S points of the QRS complex failed to be detected in only 0.60% of the beats).
(2) R-R intervals (8 features): this group consists of four features built from the original R spike annotations provided with the MIT-BIH database: the previous R-R interval, the next R-R interval, the average R-R interval in a window of 10 surrounding R spikes, and the signal mean R-R interval. The same four features are also computed using the R spikes detected by the annotation algorithm.
(3) Morphological features (19 features): ten features are derived by uniformly sampling the ECG amplitude in a window defined by the onset and offset of the QRS complex, and nine other features in a window defined by the QRS offset and the T-wave offset. As the ECG signals were already sampled, linear interpolation was used to estimate the intermediate values of the ECG amplitude. Here again, when the onset or offset points needed to compute a feature were not detected, the feature value is set to the patient's mean feature value.
(4) HBF coefficients (20 features): the parameters for computing the HBF expansion coefficients as defined in [8] are used. The order of the Hermite polynomial is set to 20, and the width parameter *σ* is estimated so as to minimize the reconstruction error for each beat.
(5) Higher-order statistics (30 features): the 2nd, 3rd, and 4th order cumulant functions are computed. The parameters as defined in [28] are used; the lag parameters range from −250 msec to 250 msec centered on the R spike, and 10 equally spaced sample points of each cumulant function are used as features, for a total of 30 features.
(6) Normalized R-R intervals (6 features): these features correspond to the same features as in the R-R interval group except that they are normalized by their mean value for each patient. These features are thus independent from the mean normal behavior of the heart of patients, which can naturally be very different between individuals, possibly misleading the classifier.
(7) Normalized segmentation intervals (21 features): this group contains the same features as in the segmentation group, except that they are normalized by their mean value for each patient. The normalization is obviously not applied to boolean segmentation features. Here again, the objective is to make each feature independent from the mean behavior of the heart of a patient, because it can naturally be very different between individuals.

Several studies have shown that using the information from both leads can increase the classification performances [4, 6]; all features are therefore computed independently on both leads (except the four R-R intervals and the three normalized reference R-R intervals computed from original annotations which are common to both leads), for a total of 249 individual features.

## 6. Experiments and Results

 For the reasons detailed in Section 4, two distinct approaches to the feature selection problem are followed, depending on the complexity of the classification model employed, a wrapper procedure with the weighted LDA model using a forward-backward search strategy and a ranking procedure with the weighted SVM model using the MI criterion.

As in heart beat classification problems around 90% of data points correspond to normal beats, a trivial model always predicting the normal class would reach an accuracy of 90%. The accuracy itself is thus not well suited for this problem and the balanced classification rate (BCR), defined as the geometric mean of class accuracies, is rather considered in this work [1]. In the above example, a BCR of only 25% would be obtained, which better reflects that only one class out of the four classes was correctly classified. According to preliminary experiments and expert opinions, the maximum number of allowed features is arbitrarily set to 10. For both models, the weights are set to the same values as in [4]: the inverse of the class priors.

**Figure 1:**
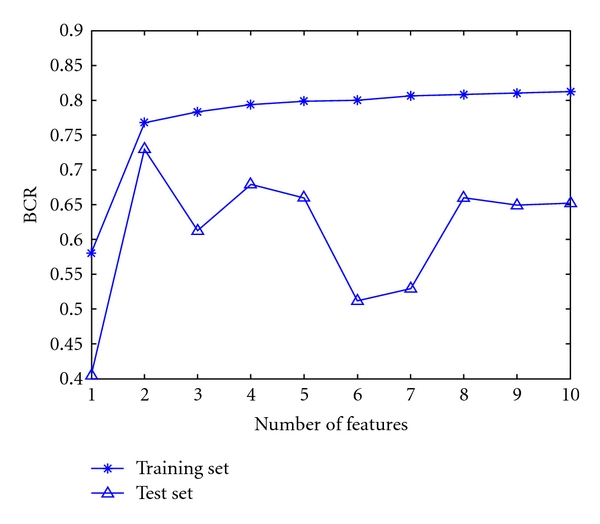
BCR obtained with the LDA and a forward wrapper feature selection procedure.

The forward-backward selection is performed on the training set and the BCR obtained at each step on both the test set and the training set is shown in Figure 1. Although a BCR of more than 80% can be reached on the training set, the best performance achieved on the test set is a BCR of 73% with only two features. These two features are the normalized value of the previous R-R interval and the high-order statistic of order one with a delay of −166 msec. Nevertheless, if the number of features was selected by taking the maximal results obtained on the training set, a BCR below 70% would be obtained on the test. Hence, the wrapper algorithm seems to overfit the training data and to generalize quite poorly.

As far as the weighted SVM model is considered, the one-against-one approach is used for multiclass classification, and the polynomial kernel is used to allow nonlinear predictions. The optimal degree of the polynomial kernel (between one and four) and the optimal value of the regularization parameter (between 10^−5^ and 10^5^) are chosen using a leave-one-patient-out cross-validation procedure on the training set. The MI value between each feature and the class labels is computed using a histogram-based estimator [14] on the training set to score the features. Following recommendations by [14], the number of bins in the histogram was heuristically chosen as the square root of *N*.

Table 3 holds the top 10 features, as ranked by the MI criterion. As it can be observed from the table, the important features seem to be R-R intervals, the amplitude and length of the T wave, and 2nd-order statistics (the autocorrelation function). The top 2 features are from patient-normalized feature sets. This is in accordance with the selection of the forward-backward algorithm with the LDA classifier since the two selected features are the first and fifth best ranked features. These findings are also in accordance with previous work such as [1], where the best performances were obtained using R-R, normalized R-R and HOS feature sets, and the second best performances with normalized interval features.

These results validate the relevance of the normalization of the features. On the other hand, several popular feature sets do not seem to serve the classification performances. No features were indeed selected by the models from the HBF coefficients, the 3rd- and 4th- order statistics, and the unnormalized segmentation intervals. Furthermore, it does not seem necessary to extract features on both leads since only features from the original annotations and from the first lead are selected.

**Figure 2:**
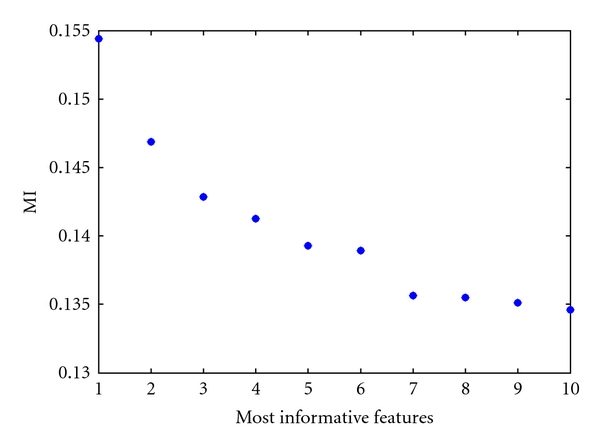
MI of the ten most informative features with the class labels.

It is important to note that unlike the correlation, the MI is not bounded, and the choice of the significantly informative features is not straightforward [15]. For this reason, and in order to keep the computational time reasonable, the number of features is chosen by looking at the sorted MI values for the 10 most informative features as shown in Figure 2. It can be observed in Figure 2 that a number of six features seem to be a reasonable choice.

Table 4 summarizes the performances achieved by the two feature selection approaches together with the performances obtained with previously reported feature choices for the same models. The classification accuracy for each class is presented, together with the BCR.

The results in Table 4 show that performing feature selection is of great importance, since the weighted SVM with only 6 features significantly outperforms all other classification procedures with up to 50 features. In particular, the accuracy for the *S* class is improved by almost 40%. This can be explained by the selection of more relevant features and by the fact that features can be chosen individually and not only at a group level. As far as LDA classification is concerned, an improvement of less than 1% of BCR can be achieved by using 50 features instead of only the 2 features selected by the wrapper method.

It is important to note that the performances reported in Table 4 are different to the ones published in [4] and in [1]. This can be explained by differences in methodologies. In [4], the authors made a tremendous work by manually correcting all the R spike annotations. Since the R-R features are clearly one of the most important features, this may explain the differences in performances. However, manually annotating all the signal is a time-consuming process which is not affordable in practice when thousands of beats have to be evaluated. The difference in performance with [1] can be explained by the fact that the authors select the hyperparameters of the SVM by measuring the performances directly on the test set rather than by using a cross-validation procedure on the training set which is a less advantageous but more realistic situation.

Eventually, it is important to note that in [7], the authors also report an increase in performance compared to [4]. However, the fusion class is merged with the *V* class in their experiments. Since the *F* class is the most unbalanced class, the classification process is then less impacted by the unbalance and likely to yield higher results. Nevertheless, when looking at the class accuracies reported in that study for the pathological classes *S* (77%) and *V* (81%) that are common to this work, our algorithm also performs better.

## 7. Conclusion

 The selection of discriminative features is known to be of great importance to help interpreting models and to increase the performances by removing spurious features. In this work, a large number of features proposed in the literature are extracted from the heart beat time series, and their relevance is evaluated on a common ground. For this purpose, two feature selection strategies are evaluated on real ambulatory recordings. The first one is an incremental wrapper procedure, and the second one is a filter approach.

The wrapper method is used with the weighted LDA model using a forward-backward search strategy. Results show that the best performances on the test set are obtained with only two features. These results are similar in terms of BCR to the performances of the same model using previously reported feature selection, were up to 50 features where required to attain the same performances.

The ranking approach is used in conjunction with the weighted SVM classifier and the MI criterion to score the features. Six features are empirically selected from the ranking procedure. Results with the weighted SVM classifier using only these 6 features are higher than previously reported interpatient algorithms.

These results show that a very small number of features are actually necessary to yield high performances and that several popular feature sets do not seem to serve the classification process. In particular, the most important features appear to be R-R intervals, the amplitude and length of the T wave, and 2nd-order statistics. Results also show that the mutual information criterion is a powerful tool for feature selection. In particular, it can be used in conjunction with models having a computational complexity which makes the wrapper procedure intractable in practice.

## Figures and Tables

**Table 1:** Grouping of the MIT-BIH-labeled heart beat types according to the AAMI standards.

Normal beats (*N*)	Supraventricular ectopic beats (*S*)	Ventricular ectopic beats (*V*)	Fusion beats (*F*)
Normal beats	Atrial premature beat	Premature ventricular contraction	Fusion of ventricular and normal beats
Left bundle branch block beats	Aberrated atrial premature beat	Ventricular escape beats	
Right bundle branch block beats	Nodal (junctional) premature beats		
Atrial escape beats	Supraventricular premature beats		
Nodal (junctional) escape beats			

**Table 2:** Distribution of heart beat classes in the two independent datasets.

	*N*	*S*	*V*	*F*	Total
Training	45809	942	3784	413	50948
	89.91%	1.85%	7.43%	0.81%	100%
Test	44099	1836	3219	388	49542
	89.01%	3.71%	6.50%	0.78%	100%

**Table 3:** Top 10 features as ranked by the MI criterion. Ref. stands for the reference annotations provided with the MIT database.

Pos.	Description	Lead
1	Previous R-R (normalized)	Ref.
2	T wave amplitude (normalized)	1
3	2nd-order statistic at −40 msec	1
4	2nd-order statistic at +40 msec	1
5	2nd-order statistic at −166 msec	1
6	2nd-order statistic at 166 msec	1
7	T wave interpolation at 50%	1
8	Previous R-R	Ref.
9	Next R-R (normalized)	Ref.
10	T wave interpolation at 60%	1

**Table 4:** Classification performances of the two feature selection methods compared to previously reported feature choices.

Model	Feature selection	Features	BCR	*N*	*S*	*V*	*F*
wLDA	[4]	50	73.83%	88.63%	44.66%	80.58%	81.44%
wLDA	Wrapper wLDA	2	73.00%	81.88%	70.53%	70.77%	68.81%
wSVM	Ranking MI	6	82.99%	75.88%	82.63%	85.06%	88.40%
wSVM	[1]	36	71.55%	77.54%	42.86%	79.19%	86.60%
